# Supplementary material for: Wood-specific modification of glucuronoxylan can enhance growth in Populus
Source: J Exp Bot. 2025 Aug 23;77(2):445–62. doi: 10.1093/jxb/eraf364 (PMC12794237; doi:10.1093/jxb/eraf364)
Supplement: eraf364_Supplementary_Data [file eraf364_supplementary_data.zip › jexbot315465-file001.pdf]

Cambium/phloem

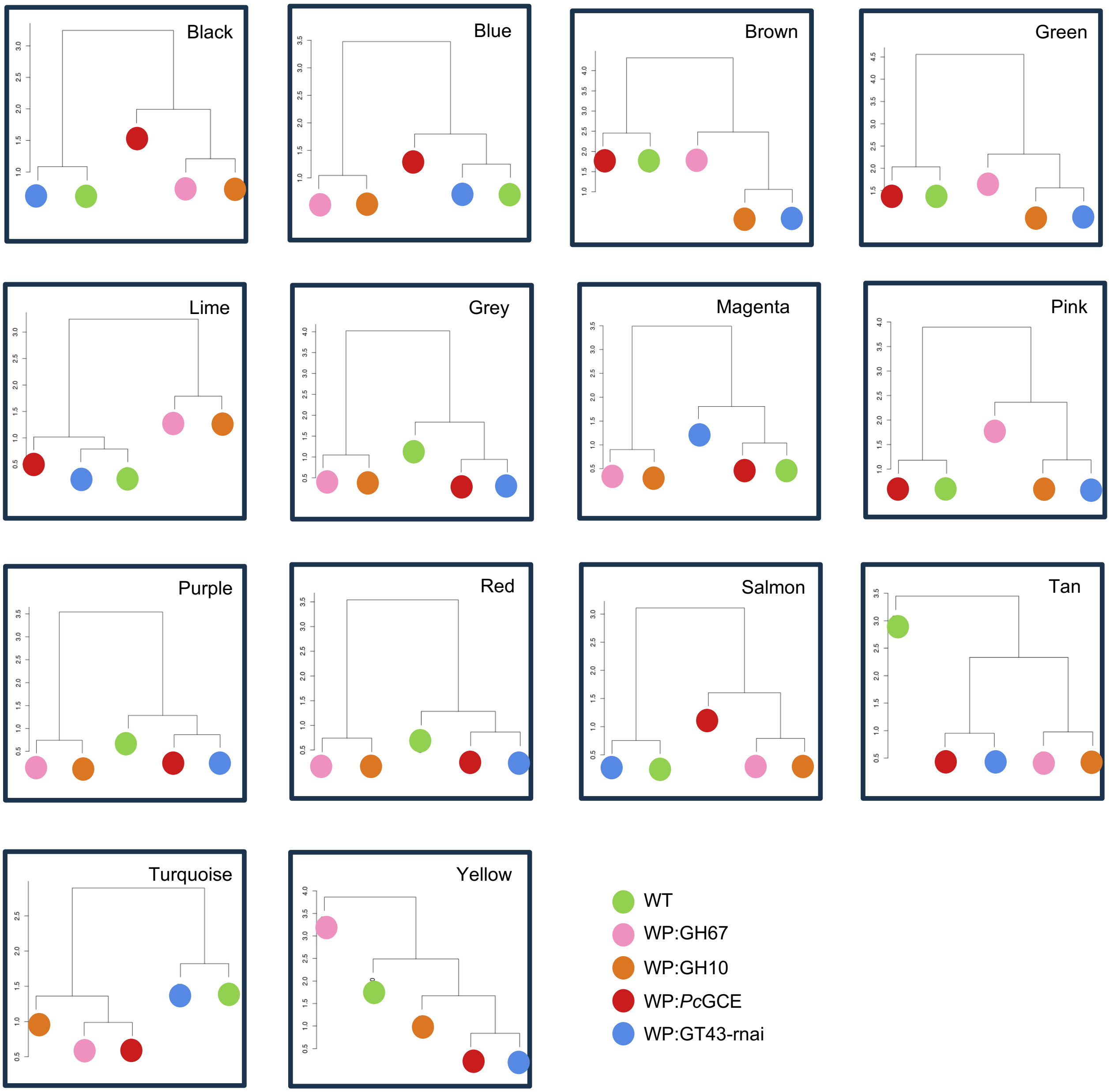

Supplementary Figure S1. Analysis of transcriptomic changes in the cambium/phloem tissues of transgenic lines compared to wild type using WGNCA. Relationships among the different genotypes in detected color-coded co-expression clusters are illustrated by dendrograms.

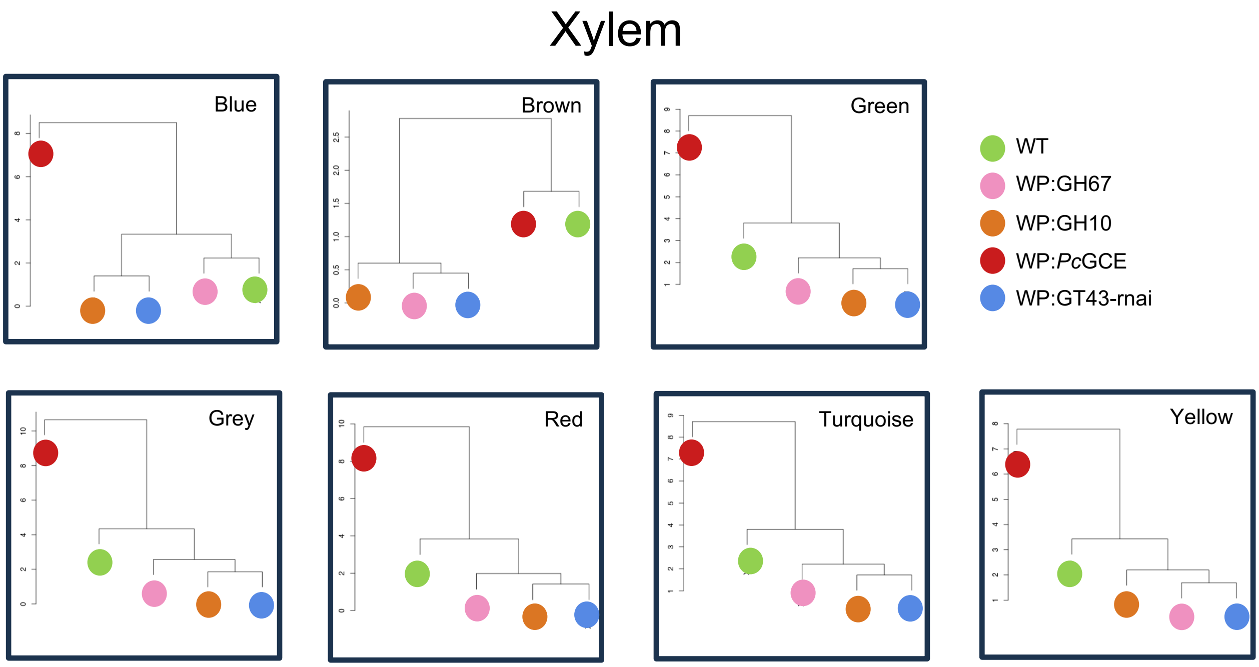

Supplementary Figure S2. Analysis of transcriptomic changes in the xylem tissue of transgenic lines compared to wild type using WGNCA. Relationships among the different genotypes in detected color-coded co-expression clusters are illustrated by dendrograms.
